# Diagnosis and Treatment of Infections in the Burn Patient

**DOI:** 10.3390/ebj5030028

**Published:** 2024-09-04

**Authors:** David G. Greenhalgh, John L. Kiley

**Affiliations:** 1 Burn Department, Shriners Children’s Northern California, 2425 Stockton Blvd., Sacramento, CA 95817, USA; 2 Department of Surgery, University of California, Davis, Sacramento, CA 95817, USA; 3 Infectious Disease Service Brooke Army Medical Center, Fort Sam Houston, TX 78234, USA; john.l.kiley.mil@health.mil; 4 Department of Surgery, Uniformed Services University of the Health Sciences, Bethesda, MD 20817, USA

**Keywords:** burns, infection, sepsis, septic shock, hospital acquired infections, antimicrobials

## Abstract

Infection is very common in burn patients because they lose the primary barrier from microorganism invasion, the skin. While there are attempts to prevent infections, topical antimicrobials and systemic prophylaxis tend to lead to more resistant organisms. After the initial resuscitation, the most common cause of death is from sepsis and multiple organ dysfunction syndrome. The diagnosis is difficult in the burn population because the constant exposure from the open wound leads to an inflammatory response that leads to persistent hypermetabolism. This paper reviews the current understanding and treatment of infection and sepsis in burns.

## 1. Introduction

Burn patients are particularly prone to infection because they lose their primary defense against microorganisms, the skin. The epidermis, along with its resident microbiome, is highly effective in limiting invasion of bacteria and fungi. Once skin is destroyed, these microorganisms have unlimited access to the underlying tissues. If the wound is superficial and small, the secondary defenses (neutrophils, monocytes, lymphocytes, and others) can handle the invasion. The epithelial cells residing in skin adnexa (hair follicles, oil glands, sebaceous glands) eventually migrate to the surface of the wound to re-create the epithelial barrier. Deeper burns lack the ability to re-epithelialize, so wounds create a temporary and less effective barrier, granulation tissue, which is filled with inflammatory cells, collagen, and new blood vessels. In full-thickness injuries, fibroblasts lay down collagen, which is used to pull the wound edges together through a process called contraction. For vulnerable areas, this wound contraction leads to contractures and hypertrophic scarring. Burns greater than 20–30% of the total body surface area (TBSA) lead to a global and profound hypermetabolic response that produces the energy required for fighting infection and closing the wound. All patients with large burns develop a systemic inflammatory response syndrome (SIRS) and are at significant risk for sepsis. Most burn patients survive their initial resuscitation, and deaths occur weeks to months later from sepsis and its corresponding multiple organ dysfunction syndrome (MODS). This review will summarize the epidemiology, diagnosis, and treatment of burn infections, and how they lead to a unique form of sepsis and MODS.

## 2. Epidemiology and Impact of Infection in Burn Patients

Compared to other patients, healthcare-associated infections (HAIs) are fairly common in burn patients. The reason for the higher incidence is related to several factors besides the obvious loss of skin. The care of major burns requires much longer hospital stays than most other diagnoses. The typical expectation is that a burn patient will have a length of stay that approximates one day per percent burn, but that number is increased in larger burns [1]. The prolonged length of stay also includes a more extended requirement for invasive lines and catheters. It is not uncommon for a patient to require a central venous catheter, arterial line, and urinary Foley catheter for weeks to even months. Alternatives to central lines, such as peripherally inserted central catheter (PICC) lines, do not eliminate central-line-associated blood stream infections (CLABSIs) [2].

Not only is the length of stay prolonged, but once a burn is greater than 20% TBSA, the persistent hypermetabolic response masks many of the signs of infection [3]. The incidence of HAIs in burns ranges from 7 to 11.7% [4,5] in recent publications from the United States and Taiwan. It is likely that numbers vary depending on location and treatment. Around the world, many burn patients never receive central lines, Foley catheters or endotracheal tubes, so CLABSIs, catheter-associated urinary tract infections (CAUTIs) or hospital- or ventilator-acquired pneumonias (HAPs, VAPs) are not relevant statistics in those settings. According to the American Burn Association’s (ABA) 2019 National Burn Repository (ABA-NBR), the most common HAI in the United States is pneumonia, which is followed by urinary tract infections (UTIs), cellulitis, wound infection, septicemia, and bacteremia [6]. It is not clear why cellulitis and wound infections are documented separately, but combined, they would outnumber pneumonias. In addition, the difference between bacteremia and septicemia would seem to be difficult to define. The ABA-NBR also compares infectious complications based on time on a ventilator (none, 1–3 days, ≥4 days). As ventilator days increase, pneumonia and septicemia rates increase, UTIs stay stable, and cellulitis/wound infections decrease [6]. Finally, the ABA-NBR reveals that infections change based on age. UTIs are more common in patients <5 and >80 years old, and pneumonias are more common in patients 5–80 years old [6].

As expected, infection has a significant impact on the outcome of any burn patient. In 2009, Herndon’s group reported that the leading cause of death in pediatric burn patients was sepsis (47%) and that the rate increased from 35% in the first 10 years of the study to 54% in the next 10 years [7]. In 2019, the Loyola, Illinois burn center reported that 39% of their patients admitted to the intensive care unit with burns ≥ 10% TBSA developed sepsis [8]. Of the 39%, 18.9% developed septic shock. The overall mortality rate of the patients with sepsis was 13.3%, but it was only 3.7% with sepsis alone and 49% with septic shock. Sepsis and/or septic shock led to seven times increased odds of mortality (odds ratio 7.04, 95% confidence interval 1.93–25.7) compared to patients with no sepsis [8]. Several other studies indicate that sepsis leading to MODS is the leading cause of death in burn patients [9,10,11,12,13]. Clearly, infection, which often leads to sepsis, has a profound influence on the outcomes of burn patients.

## 3. Infection versus Systemic Inflammatory Response Syndrome (SIRS)

Infection is a local invasion of a microorganism, whether bacterial, fungal, viral, or other. The response to infection starts locally and is manifested by typical signs of inflammation as described by Celsus in the second century (*rubor*, *calor*, *dolor* and *tumor*) [14]. The pathophysiology of the local response to infection is now well known [15,16]. The tissues are full of resident macrophages whose role is to detect foreign proteins or signs of damage and attempt to return the area to homeostasis [17]. These inflammatory cells have receptors, called pattern recognition receptors (PRRs), that detect components of the invading organisms. These by-products, called pathogen-associated molecular patterns (PAMPs), bind to the receptors to induce intracellular signaling that leads to the production of proteins (cytokines) that attract inflammatory cells into the site of infection. The classic pathway is characterized by the production of the Gram-negative bacterial wall molecule, lipopolysaccharide (LPS), which, in simplest terms, binds to Toll-like receptor-4 (TLR4) to initiate signaling through the NF-κB pathway to produce multiple cytokines, especially tumor necrosis factor-α (TNFα), interleukin-1 (IL-1), and interleukin-6 (IL-6). These cytokines, plus many more, alter the nearby capillary endothelial cells to increase leukocyte adhesion, attract neutrophils and other inflammatory cells, and initiate local inflammation. There are 10 human TLRs in humans and multiple other PRR systems (RIG-1, MDA5, NOD1, NOD2, STING, RAGE and many more—see references [15,16,17]). The response is local vasodilation (*rubor*), increased heat because of the vasodilation (*calor*), increased capillary leak (*tumor*), and pain from the local mediators (*dolor*). The inflammatory cells are called to the area by chemotaxis (following the cytokine concentration gradient), adhere to the capillary bed and migrate into the area to release their “weapons” to destroy the invaders. Fibroblasts are also called to lay down an extracellular matrix to wall off the area so that the organisms cannot escape. As more neutrophils invade the area, they die off and create an abscess, which when drained eliminates the infection.

When the cytokine release is large enough, there is a response called systemic inflammatory response syndrome (SIRS) [14,15,16,17]. The cytokines are detected in the hypothalamus, which sends signals to the pituitary gland to signal the medulla of the adrenal gland to release catecholamines. The catecholamine release leads to tachycardia, tachypnea, and fever. At the same time, adrenocorticotropic hormone (ACTH) is released to increase cortisol release that assists with the breakdown of muscle to provide fuel to assist with fighting infection and wound healing [18]. Since the primary barrier to microbial invasion is lost in burns, the release of catecholamines persists for weeks to months. The chronic exposure to the hostile environment leads to persistent changes in the white blood cell count, so leukocytosis or leukopenia is not uncommon. All these factors led to the agreement that all patients with large burns have SIRS [19]. Many hospitals have “SIRS Alerts” that lead to automatic actions to rule out infection (e.g., measure serum lactate and obtain cultures), but it is clear that these alerts are irrelevant for burn patients.

## 4. Burn Sepsis versus Standard Sepsis

Sepsis has been defined by the Sepsis-3 group (convened by the Society of Critical Care Medicine and the European Society of Intensive Care Medicine) as “life-threatening organ dysfunction caused by a dysregulated response to infection” [20,21,22]. While this “concept” definition is appropriate for all types of sepsis, it is really a concept and not a usable definition. The same Sepsis-3 group developed the “practical” definition that sepsis is “suspected or documented infection and an acute increase of ≥2 SOFA (Sequential Organ Failure Assessment) points (a proxy for organ dysfunction)”. The same publication defined septic shock as “a subset of sepsis in which underlying circulatory and cellular/metabolic abnormalities are profound enough to substantially increase mortality”. The “practical” definition of septic shock is “a clinical construct of sepsis with persistent hypotension requiring vasopressors to maintain mean arterial pressure (MAP) ≥ 65 mmHg and having a lactate level > 2 mmol/L (millimoles/liter) or 18 mg/dL (milligrams/deciliter) despite adequate volume resuscitation. With these criteria, hospital mortality is in excess of 40%”.

It is clear that early recognition and treatment of sepsis can improve patient survival [23,24,25]. In an effort to improve the recognition of sepsis, and to improve its treatment, there have been four Surviving Sepsis Campaigns that have produced early diagnostic criteria along with bundles that should be completed within three or six hours of diagnosis [26,27,28,29]. While these definitions and guidelines are useful for all forms of sepsis and septic shock, it is important to recognize that there are many differences between sepsis in burns and sepsis in the general population [12]. Most patients with sepsis present to the emergency department or occupy a hospital bed when they develop an acute change in their status suggesting an infection. Sepsis is the primary diagnosis, or it occurs relatively early in their hospital course. The classic early systemic signs are typical of SIRS: fever, tachycardia, tachypnea, and leukocytosis. Therefore, early diagnosis and treatment is relatively easy. Unlike other patients, burn patients have lost the primary barrier to microbial invasion, their skin. The diagnosis of sepsis in burns is more difficult and often occurs days, weeks, or even months after admission. Since the burn patient develops a persistent hypermetabolic response to the injury, fevers, tachycardia, tachypnea, and changes in white blood counts are almost always present. As stated earlier, their prolonged stay leads to prolonged exposure to invasive lines and catheters which further increases their risk of infection.

In an attempt to deal with these differences, in 2006, the ABA led a consensus conference to define the definitions of sepsis and infection in burn patients [19]. They declared that all burns > 20% TBSA have SIRS. In addition, the trigger to initiate the treatment of burn sepsis was changed to address the unique issues in burn patients (Table 1).

They agreed to raise the temperature of a fever to 39 °C and increased the criteria for tachycardia and tachypnea. Other factors were mentioned that also raise the concern for sepsis such as thrombocytopenia, hyperglycemia, feeding intolerance and diarrhea. The ABA Consensus criteria have been challenged by several well-designed studies that suggest that other indicators should be used for the diagnosis and treatment of burn sepsis [30,31,32]. These criteria should be challenged and tested. The most recent effort to address burn sepsis occurred at the 2022 International Society for Burn Injuries (ISBI) meeting in Guadalajara, Mexico. There was a meeting of burn caregivers representing experts from around the world to develop a Surviving Sepsis After Burn Campaign. The goal was to develop diagnostic and early treatment criteria for burns modeled after the current Surviving Sepsis Campaign. The goal will be to develop the criteria for burn sepsis as a guideline to create multicenter trials to test and revise the criteria. The Surviving Sepsis After Burn Campaign, published in 2023, should provide the first step for improving the treatment of infection and sepsis in burn patients [33].

## 5. Types and Management of Infections in Burn Patients

### 5.1. Burn Wound Infections

Although the burn wound has lost its barrier to the outside, actual infection of the burn wound is relatively uncommon in the modern era, particularly in developed countries. The wound is rapidly contaminated with organisms, but with simple cleansing with soap and water, the risks for destructive infections are diminished. The classic teaching suggests that all burn wounds need to have a topical antimicrobial ointment to reduce infections [34,35,36]. It is not clear, however, if the topical antimicrobials prevent infection or simply select out more resistant organisms [37]. When one looks at the microorganisms that are cultured from the wound, the first organisms to be found are *Streptococcus* and *Staphylococcus* species. Therefore, many burn centers treat small, superficial wounds with an ointment such as bacitracin (which covers only Gram-positive bacteria). If the wound remains open for prolonged periods, then Gram-negative organisms appear, with *Pseudomonas aeruginosa* becoming quite common. Therefore, deeper wounds are often treated with an ointment or cream that has broader coverage (such as silver sulfadiazine). As time progresses, wounds often will have yeast or even fungal colonization. At the later timepoints, multi-drug-resistant organisms (such as *Klebsiella*, *Acinetobacter*, *Stenotrophomonas* and *Pseudomonas* species) tend to be found in the wounds [38]. One question is: are we selecting out resistant organisms with our topical antimicrobials, or would the bacteria evolve without our treatments? It is clear, however, that providing systemic antibiotics does select out resistant organisms [4,39,40,41]. Prophylactic systemic antibiotics should be avoided in the treatment of burn patients!

The major reason why burn wound infections have decreased is that burn caregivers are much more aggressive with the surgical treatment of larger and deeper burns. Superficial wounds seem to do well with soap and water and dressings that protect the wound as it re-epithelializes. Clearly deep burns are also treated more aggressively. Decades ago, third-degree (full thickness) burns were treated expectantly with topical agents and debridement of loose tissue. Eschar is the thick layer of dry material, that is the result of heat-induced protein changes of the skin. Caregivers would wait for the eschar to separate from the underlying granulation tissue. It turned out that eschar separation is the result of bacterial proliferation and their enzymes breaking down the proteins at the interface with viable underlying tissue. The body would create granulation tissue (the red, weeping, vascular tissue of open wounds) to act as a mediocre barrier to invasion. It would not be uncommon for an abscess to form between the eschar the wound bed, so infection rates were fairly high. Burn wound infections have been greatly reduced since the treatment of deep burns has changed completely to include early excision and grafting [42,43,44].

While greatly reduced, burn wound infections still occur. Since wounds are always colonized, positive cultures do not necessarily mean that the wound is infected. One must examine the burns on a regular basis, and the diagnosis of burn wound infection includes changes in the appearance of the wound. The wound may have a new exudate, expanding erythema, or significant changes in color. To assist with the diagnosis, the 2007 ABA Consensus conference proposed definitions of burn wound infections (Table 2) [19].

The most common infection in superficial wounds is cellulitis. Cellulitis was defined as advancing erythema, induration, warmth, and tenderness that surrounds a wound. In addition, bacteria should be present in high concentrations in the wound or burn eschar. These infections tend resolve quickly with antibiotics that cover Gram-positive organisms. On occasion, burn wounds or split-thickness donor sites (which are surgeon-induced partial thickness wounds) break down despite being re-epithelialized. This finding suggests a *Staphylococcus aureus* infection, since certain species of *Staphylococcus* (usually methicillin sensitive) produce an enzyme that can break down the bonds holding epithelial cells to each other and to the wound [45]. The wounds typically improve with a cephalosporin such as cephalexin or cefazolin. A similar sign of infection is when a superficial burn “converts” to become a full thickness wound. Failure of skin graft take is another indication of an infection. The graft will “melt” away and frequently is surrounded by purulence. The classic teaching has been that when the concentration of bacteria in a wound biopsy is >10^5^ organisms/gram, the risk of infection is very high [35,46,47,48]. This concept is probably true when a skin graft is lost, but since burns are excised so much earlier than in the past, tissue biopsies are rarely obtained [47].

**Figure 1:**
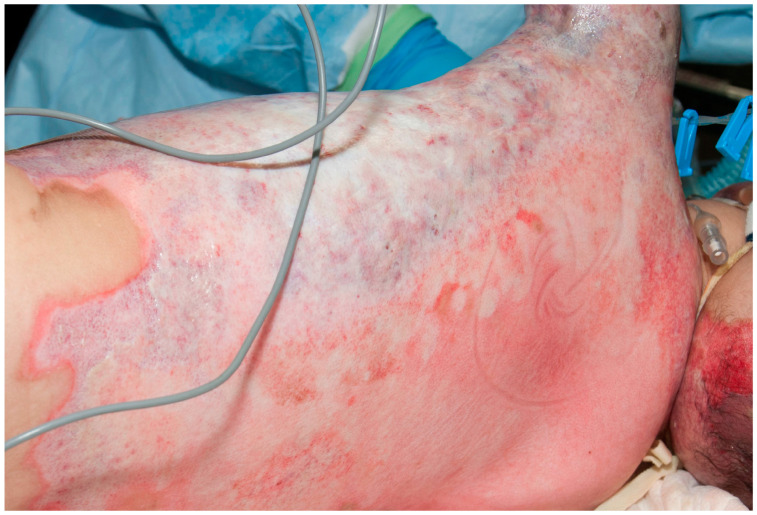
This child had partial thickness burns that developed the classic purple–gray appearance of invasive *Pseudomonas aeruginosa*. She developed profound septic shock and died the next day.

There are some specific invasive wound infections that can lead to significant problems in the burn patient. Again, their diagnoses are dependent on significant changes in the burn wound. One of the most devastating invasive infections is caused by *Pseudomonas aeruginosa* [49]. While *Pseudomonas aeruginosa* is a common colonizer of burn wounds, especially after the second week of hospitalization, coming from the digestive tract of the patient or from cross-contamination from healthcare personnel, on occasion, it can invade the wound to destroy tissue and leads to profound septic shock. Typically, *Pseudomonas aeruginosa* will leave a harmless yellow exudate in the wound. When *Pseudomonas aeruginosa* invades, the wound often develops a purple to gray color; superficial burns, or even split-thickness donor sites, become full thickness (Figure 1).

At the same time, the patient develops profound septic shock. The treatment is aggressive excision of all of the wounds and systemic treatment with anti-Pseudomonal antibiotics. Even with aggressive treatment, patients with minor, superficial wounds may not survive. This problem prompted the development of mafenide acetate as a topical antimicrobial cream [50]. Mafenide acetate will penetrate the wound and is highly effective against *Pseudomonas aeruginosa*. Silver sulfadiazine combined with cerium nitrate in a cream formulation also penetrates burn eschar, prevents the proliferation of microorganisms, and is less painful than mafenide acetate and is used widely in European countries.

**Figure 2:**
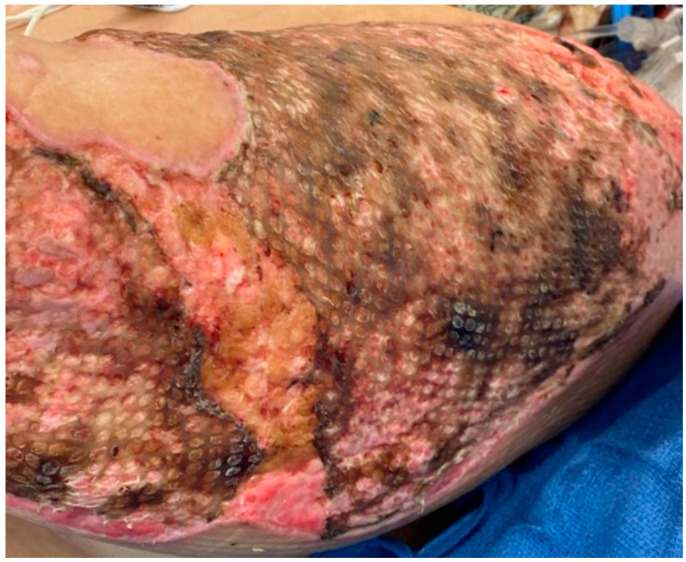
This wound has the whitish-brown color of *Aspergillus* in the recently grafted burn. The mold developed under the graft and eventually there was complete loss of the autograft.

**Figure 3:**
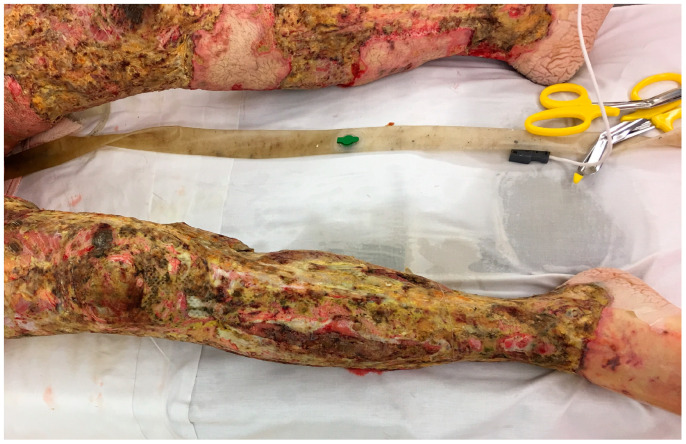
This man developed invasive *Fusarium* infection throughout his grafted burns. Despite multiple excisions, the mold continued to return, and he died.

Infections from *Candida* species are not very common in the burn wound, but they can occur and tend to act more like the bacterial infections [51]. They may be selected by antimicrobials that do not cover yeast, such as bacitracin or mafenide acetate. On occasion, the topical use of nystatin or miconazole is needed. Invasive fungal infections, often with filamentous fungi, while less common than bacterial infections, do occur in patients with large burns [51,52,53,54]. In some cases, a small section of nonviable fat (such as in the interstice of a widely meshed autograft) will develop a soft white exudate that looks like cottage cheese. The most common fungus is *Aspergillus*, but Mucorales species or *Fusarium* may also invade the wound. It is not uncommon for the fungi to spread and destroy the surrounding grafts (Figure 2 and Figure 3).

The treatment involves re-excision of the infected wound and broad-spectrum systemic antifungals. Topical application of amphotericin B has been described with limited success.

**Figure 4:**
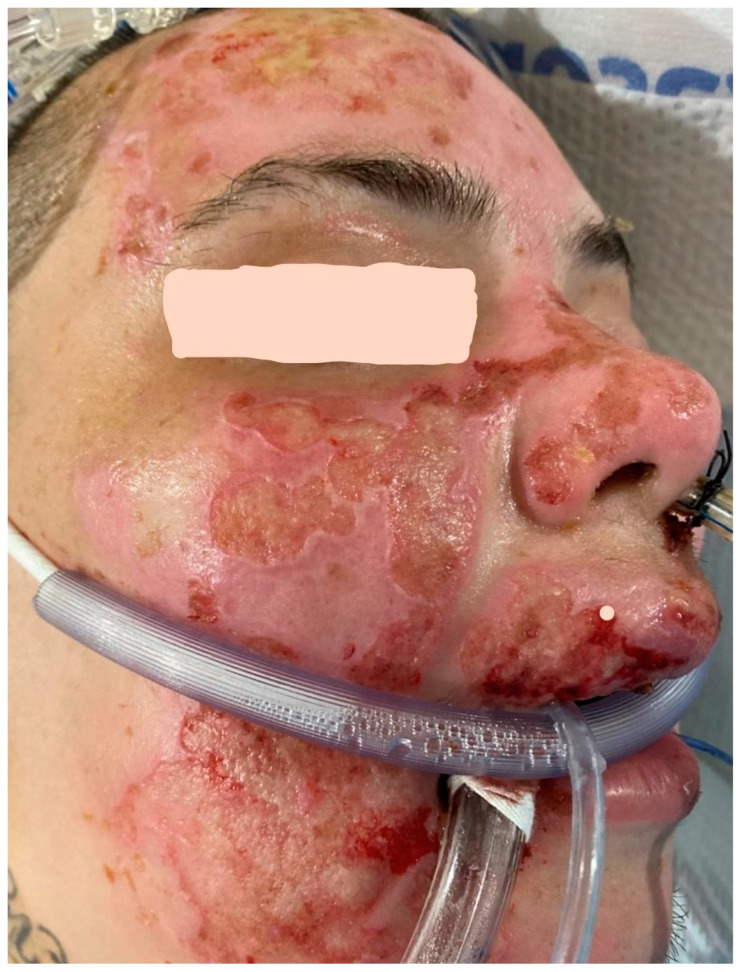
This patient has the classic “punched-out” lesions of *Herpes simplex* 1 infection in the previously healed second degree burns of the face. The wounds healed after treatment with systemic acyclovir.

Another relatively common infection of superficial wounds, especially in face burns, results from the activation of Herpes viruses such as HSV-1, HSV-2, or VZV [55,56,57,58]. These infections are easy to diagnose based on physical examination. A healed or nearly healed wound will develop punched out lesions that can spread to destroy a significant part of the epithelium (Figure 4).

Viral cultures will confirm the diagnosis, but starting acyclovir or ganciclovir based on the examination will usually resolve the problem. Rarely, *Varicella* (chicken pox) can produce similar lesions in burns [59]. *Varicella* can cause significant damage to healing wounds but is also readily treatable with similar antiviral medications.

Other infecting syndromes that play important roles in morbidity in burn patients including pneumonia often develop in the weeks post-burn resuscitation. While traditional risk factors for the development of pneumonia in these patients are included in the pathophysiology of these syndromes in burn patients (e.g., prolonged ventilatory requirements, prolonged hospital stay), there are also interesting questions about the role the changing microbiome after burn injury has in the microbiologic etiology of these infecting syndromes [60].

### 5.2. Sepsis and Septic Shock

As stated earlier, the diagnosis and treatment of sepsis and septic shock is different in the burn patient when compared to the general population. Sepsis tends to occur days, weeks or even months after the original injury. The cause of sepsis in burns is not always clear. Invasive wound infections (especially invasive *Pseudomonas aeruginosa*) occasionally will cause sepsis. More commonly, iatrogenic causes such as CLABSIs, pneumonias or UTIs are responsible for the infections [61]. Clearly, with new signs of sepsis, invasive catheters should be replaced. It is not uncommon to have early signs of sepsis resolve with the replacement of a central line. There are many times when no obvious sources of sepsis are found in the burn patient. Even more frustrating is the fact that fatal sepsis can occur months after the original burn even when the patient’s wounds are covered. Sepsis, along with the stress of the hypermetabolic response, may lead to sequential organ failure. Most late deaths are related to multiple organ dysfunction syndrome (MODS) [9,10,11,12,13].

The early diagnosis of sepsis requires close attention by the caregiver. Rounding on critically ill burn patients should take place at least daily and preferably twice a day. Many times, subtle indicators of impending sepsis are missed unless close attention is given to the patient. The early signs of burn-related sepsis are increased fluid requirements, decreased urine output, confusion, feeding intolerance, diarrhea, hyperglycemia, increased insulin requirements and high fever (>39.0 °C) [19,62]. The white blood cell count may dramatically rise or fall, but it is a very nonspecific finding. Leukocytosis is very common throughout the hospital stay. A more sensitive indicator is a dropping platelet count [62,63,64]. Since platelets are often consumed as perfusion to capillary beds decreases and they thrombose, their numbers drop precipitously. Later in the course, this ongoing micro-thrombosis may also lead to disseminated intravascular coagulation (DIC). Biomarkers such as procalcitonin can be used as well but should not be interpreted in isolation. As the Surviving Sepsis after Burn Campaign suggests if a combination of these findings are identified, cultures should be obtained, wounds should be checked, lines should be changed, and broad-spectrum antibiotics should be initiated. Certain organisms, especially *Klebsiella*, can lead to profound septic shock within hours, so delaying antibiotic coverage is inappropriate. Sepsis and septic shock produce a systemic capillary leak syndrome that can lead to significant edema. On occasion, septic burn patients may also develop abdominal compartment syndrome, which may eventually require a laparotomy [65,66]. The treatment of septic shock is supportive with fluid resuscitation and then vasopressors (norepinephrine is preferred) to maintain an MAP ≥ 65 mmHg. In essence, the treatment of septic shock is similar to that of any other patient. There are guidelines for treating sepsis and septic shock in the Surviving Sepsis Campaign [29]. The new Surviving Sepsis After Burn Campaign should provide a new tool for treating sepsis and septic shock in burn patients [33].

### 5.3. Combat Trauma Related Burns and the Rising Epidemic of Multi-Drug-Resistant Organisms

With the invasion of Ukraine in February 2022, the world was reminded yet again that combat trauma and the subsequent development of infections related to these injuries is never far from actualization. Given the nature of modern warfare, burns suffered predominantly secondary to blast injury are likely to continue be a feature of combat injuries. Prior to the war in Ukraine, the U.S. and its allies have published extensively on hard-won lessons learned while managing combat-related burns and subsequent morbidity. These lessons included the importance of Gram-negative infections acquired through the healthcare chain, risk factors for IFI such as injuries requiring massive transfusion, the key role that infection prevention and control played in prevention of infections, as well as the high morbidity associated with combat burns [67,68]. The current conflict in Ukraine microbiologically has been dominated by New-Dehli metallo-beta-lactamse (NDM) producing *Klebsiella pneumoniae*, which is a unique challenge, as it is often extensively drug resistant if not pan-drug resistant [69,70,71,72]. Sophisticated whole-genome sequencing analyses have shown that these microorganisms have already spread across the European continent. Expertise in managing these types of burn infections, as well as new strategies to break the cycle of acquisition and transmission, will continue to be desperately needed for the future.

## 6. Conclusions

The treatment of infections and sepsis in burn patients has made a lot of progress. Despite successes, sepsis is the ultimate cause of death in burn patients. Treatment of infection and sepsis in burn patients is unique from the typical patient. Progress has been made to identify and treat burn-specific sepsis. Despite these advancements, prevention and treatment will be an ongoing effort to keep up with the ongoing increase in resistance to our current treatments.

## Figures and Tables

**Table 1:** The American Burn Association consensus definition of sepsis.

Temperature > 39 °C or <36.5 °C
Progressive tachycardia > 110 beats per minute (adults)
Progressive tachypnea > 25 breaths per minute or minute ventilation > 12 L/min (adults)
Thrombocytopenia < 100,000/µL (does not apply until 3 days after burn) (adults)
Hyperglycemia in the absence of pre-existing diabetes mellitus
(Untreated plasma glucose > 200 mg/dL or insulin resistance indicated by >7 units of insulin/h IV drip or >25% increase in insulin requirements over 24 h)
Inability to continue enteral feedings > 24 h
(Abdominal distension, enteral feeding intolerance [two times feeding rate in adults], uncontrollable diarrhea [>2500 mL/day])
In addition, it is required that a documented infection is identified as follows:
Culture-positive infection or
Pathologic tissue source identified or
Clinical response to antimicrobials

Based on the 2007 American Burn Association Consensus Conference [19].

**Table 2:** The American Burn Association consensus definitions of burn wound infection.

Burn wound colonization
Bacteria present on the wound surface at low concentrations.
Pathologic diagnosis: <10^5^ bacteria/gram tissue. *
Wound colonization occurs in all wounds and may be evident by an exudate or swab culture but the wounds are not infected.
Burn wound infection (BWI)
Bacteria present in the wound and wound eschar at high concentrations.
Noninvasive BWI
Pathologic diagnosis: >10^5^ bacteria/gram tissue. *
No signs of invasion of unburned or viable skin/tissue.
Invasive BWI
“Presence of pathogens in a burn wound at concentrations sufficient in conjunction with depth, surface area involved, and age of patient to cause suppurative separation of eschar or graft loss, invasion of adjacent unburned tissue or cause the SIRS of sepsis.”
Pathogen is typically present in the wound at high concentrations.
Pathologic diagnosis: >10^5^ pathogens/gram tissue. *
Invasion or destruction of unburned skin/tissue.
Invasive infection may occur with or without sepsis

Based on the 2007 American Burn Association Consensus Conference [19]. * Note that quantitative cultures are rarely used.
